# Hemoglobin mRNA Changes in the Frontal Cortex of Patients with Neurodegenerative Diseases

**DOI:** 10.3389/fnins.2018.00008

**Published:** 2018-01-22

**Authors:** Silvia Vanni, Marco Zattoni, Fabio Moda, Giorgio Giaccone, Fabrizio Tagliavini, Stéphane Haïk, Jean-Philippe Deslys, Gianluigi Zanusso, James W. Ironside, Margarita Carmona, Isidre Ferrer, Gabor G. Kovacs, Giuseppe Legname

**Affiliations:** ^1^Laboratory of Prion Biology, Department of Neuroscience, Scuola Internazionale Superiore di Studi Avanzati, Trieste, Italy; ^2^Neurology and Neuropathology Unit, Istituto di Ricovero e Cura a Carattere Scientifico Foundation Carlo Besta Neurological Institute, Milan, Italy; ^3^UPMC Univ Paris 06 UMR S 1127 and Centre National de la Recherche Scientifique UMR 7225, ICM, Sorbonne Universités, Paris, France; ^4^Atomic Energy Commission, DRF, Jacob, SEPIA, Fontenay-aux-Roses, France; ^5^Department of Neurosciences, Biomedicine and Movement Sciences, University of Verona, Verona, Italy; ^6^National CJD Research and Surveillance Unit, Centre for Clinical Brain Sciences, University of Edinburgh, Edinburgh, United Kingdom; ^7^Department of Pathology and Experimental Therapeutics, University of Barcelona, Barcelona, Spain; ^8^Institut d'Investigació Biomédica de Bellvitge (IDIBELL), Bellvitge University Hospital (CIBERNED), Hospitalet de LLobregat, Spain; ^9^Institute of Neurology, Medical University of Vienna, Vienna, Austria

**Keywords:** hemoglobin, prion, RT-qPCR, Alzheimer's disease, vCJD, neurodegeneration

## Abstract

**Background:** Hemoglobin is the major protein found in erythrocytes, where it acts as an oxygen carrier molecule. In recent years, its expression has been reported also in neurons and glial cells, although its role in brain tissue remains still unknown. Altered hemoglobin expression has been associated with various neurodegenerative disorders. Here, we investigated hemoglobin mRNA levels in brains of patients affected by variant, iatrogenic, and sporadic forms of Creutzfeldt-Jakob disease (vCJD, iCJD, sCJD, respectively) and in different genetic forms of prion diseases (gPrD) in comparison to Alzheimer's disease (AD) subjects and age-matched controls.

**Methods:** Total RNA was obtained from the frontal cortex of vCJD (*n* = 20), iCJD (*n* = 11), sCJD (*n* = 23), gPrD (*n* = 30), and AD (*n* = 14) patients and age-matched controls (*n* = 30). RT-qPCR was performed for hemoglobin transcripts *HBB* and *HBA1/2* using four reference genes for normalization. In addition, expression analysis of the specific erythrocyte marker *ALAS2* was performed in order to account for blood contamination of the tissue samples. Hba1/2 and Hbb protein expression was then investigated with immunofluorescence and confocal microscope analysis.

**Results:** We observed a significant up-regulation of *HBA1/2* in vCJD brains together with a significant down-regulation of *HBB* in iCJD. In addition, while in sporadic and genetic forms of prion disease hemoglobin transcripts did not shown any alterations, both chains display a strong down-regulation in AD brains. These results were confirmed also at a protein level.

**Conclusions:** These data indicate distinct hemoglobin transcriptional responses depending on the specific alterations occurring in different neurodegenerative diseases. In particular, the initial site of misfolding event (central nervous system vs. peripheral tissue)—together with specific molecular and conformational features of the pathological agent of the disease—seem to dictate the peculiar hemoglobin dysregulation found in prion and non-prion neurodegenerative disorders.

In addition, these results suggest that gene expression of *HBB* and *HBA1/2* in brain tissue is differentially affected by distinct prion and prion-like aggregating protein strains. Validation of these results in more accessible tissues could prompt the development of novel diagnostic tests for neurodegenerative disorders.

## Introduction

Neurodegenerative diseases are incurable debilitating disorders of the nervous system, characterized by structural and functional neuronal loss. Approximately 30 million people are affected worldwide, and this number is predicted to reach more than 100 million by 2050 (Benetti et al., 2012).

These disorders, which include Alzheimer's, Parkinson's, and prion diseases among others, are characterized by the accumulation of aggregated proteins in the form of amyloid, often associated to pathological lesions (Jucker and Walker, 2013). The aggregation of amyloidogenic proteins can result either in a gain of toxic functions, derived from the damage provoked by these deposits in affected tissue, or in a loss of functions, due to the sequestration and the consequent inability of the aggregating protein to ensure its physiological role.

Prion diseases etiology can be sporadic, infectious or genetic, with the various forms of Creutzfeldt-Jakob Disease (CJD)—sporadic, familial, variant, and iatrogenic—being the most common prion disorder in humans. Genetic diseases include Gerstmann–Straussler–Scheinker (GSS) and Fatal Familial Insomnia (FFI). Pathological hallmarks include spongiform vacuolation in brain tissue, neuronal loss, microglial activation and astrocytes proliferation, together with the intracerebral accumulation of the protease-resistant form of the prion protein (PrP^Sc^).

More than three decades ago, globin-related mRNAs were reported in the mouse brain (Ono and Cutler, 1978). In 2009, the finding of α and β hemoglobin transcripts in *substantia nigra* neurons of mesencephalic dopaminergic cell system (Biagioli et al., 2009) opened the doors to an unexplored field of research. In particular, hemoglobin expression in mice and human neurons has been validated both at transcriptional and protein levels (Richter et al., 2009; Brown et al., 2016), while its glial expression remains unclear (Richter et al., 2009; Schelshorn et al., 2009; Chuang et al., 2012). Indeed, a decrease, sometimes an absence, of hemoglobin expression in neurons containing neurofibrillary tangles (NFTs), pre-tangles, and α-synuclein deposits in Alzheimer's disease (AD), argyrophilic grain disease (AGD), and dementia with Lewy bodies (DLB) brains, respectively, has been reported (Ferrer et al., 2011).

The function of neuronal hemoglobin (nHb) is still debated. Its ability to bind molecular oxygen is effective for the precise regulation of O_2_ homeostasis required in neurons (Ohyagi et al., 1994). In particular, while erythrocyte hemoglobin is known to be responsible of O_2_ delivery to body tissues, its brain tissue expression could be related to facilitation of O_2_ uptake into neurons, acting as an oxygen capacitor molecule (Schelshorn et al., 2009). The role of Hb in neuronal oxygen homeostasis is supported by the finding that its canonical α_2_β_2_ tetrameric structure, necessary for O_2_ coordination, is maintained also in neurons (Russo et al., 2013).

Neurons are metabolically active cells, therefore mitochondria are essential for their physiology. Hb involvement in mitochondrial respiration and regulation of the redox system is supported by several findings in literature (Biagioli et al., 2009; Brown et al., 2016). In particular, a strong down-regulation of hemoglobin α, adult chain 2, and β chains mRNA has been reported in neurons of rat treated with rotenone, an inhibitor of complex 1 of the mitochondrial respiratory chain (Richter et al., 2009).

Since iron is essential for hemoglobin oxygen transport, altered neuronal levels of α and β chains corroborates the hypothesis of a disruption in iron homeostasis, found in sCJD, AD, and PD brains, as one of the key feature at the basis of neurodegeneration (Singh, 2014).

Thanks to iron-containing heme, Hb is able to interact also with amyloid-β (Aβ), promoting its aggregation (Wu et al., 2004; Chuang et al., 2012). Double immunofluorescent assay on AD brain patients, revealed co-localization of hemoglobin and Aβ in senile plaques and cerebral amyloid angiopathy, suggesting hemoglobin involvement in AD pathology (Wu et al., 2004).

In prion diseases, some evidence exists for hemoglobin involvement in different animal models. Hba-a1 and Hbb-y were found down-regulated both in the preclinical and clinical phase in the CNS of scrapie-infected mice (Booth et al., 2004a). Furthermore, down-regulation of *HBB* and *HBA2* expression levels in late stage of intracranially BSE-infected macaques (Barbisin et al., 2014) and *HBA2* down-regulation in the blood of preclinical atypical BSE-infected cattle (Xerxa et al., 2016) suggest an involvement of hemoglobin genes in the host response to BSE prion strain infection.

In the present study, we decided to investigate the hemoglobin genes expression in a wide variety of human prion diseases, with the aim to shed light on the role of different prion strains and routes of infections on the hemoglobin regulation.

## Materials and methods

### Ethics statement

All samples were collected according to the ethical and safety regulations of the Countries in which samples were collected, and with the approval of the local ethics committees.

Human biological samples and associated data were obtained from the Austrian CJD Surveillance and KIN-Biobank Medical University of Vienna (Austria), the MRC Edinburgh Brain Bank (UK), the Institute of Neuropathology Brain Bank (HUB-ICO-IDIBELL Biobank) (Barcelona, Spain), the French National Neuropathological Network for CJD and the French National Centre of Reference for prions (France), the Carlo Besta Neurological Institute (Milan, Italy) and the University hospital of Verona (Italy).

Informed and written consent for the research use of autopsy tissue was obtained from the relatives of the deceased whenever necessary in accordance with the Declaration of Helsinki (1964–2008) and the Additional Protocol on the Convention of Human Rights and Biomedicine concerning Biomedical Research (2005). *PRNP* gene was analyzed pre-mortem in all cases with the informed and signed consent of the patient's family.

### Patient samples

The study was performed on samples coming from the following cases: AD (*n* = 14) Braak stages I-III, sCJD (*n* = 23), genetic prion diseases gPrD (*n* = 30), vCJD (*n* = 20), and iCJD (*n* = 11). Age-matched subjects died from unrelated conditions and lacking any neurological sign and with no pathological lesions in brain were included as controls (*n* = 30). Prion disease cases were all confirmed by means of Western Blot and/or *PRNP* sequencing, while AD diagnoses were confirmed through neuropathological analysis.

The full list of samples and patient details is reported elsewhere (Vanni et al., 2017). Blood samples (*n* = 4) were obtained by Carlo Besta Neurological Institute (Milan, Italy).

### Tissue and RNA extraction

Total RNA from frozen frontal cortex was extracted with PureLink RNA Mini Kit (Life Technologies) and on-column DNA digestion. Blood samples were collected in PAXgene Blood RNA Tubes (Qiagen) and the related RNA was extracted using PAXgene Blood RNA Kit (Qiagen). RNA integrity was analyzed using 2100 Bioanalyzer (Agilent Technologies). Samples included in our gene expression analysis displayed RNA Integrity Number (RIN) ≥ 4, with the exception of few rare samples.

### Reverse transcription and real time-quantitative PCR (RT-qPCR)

For frontal cortex, cDNA was obtained from 3 μg of total RNA with 50 μM Oligo(dT)20, 10 mM dNTP mix, 5X First Strand Buffer, 0.1 M DTT, 40 U RNAse inhibitor, and 200 U SuperScript® III Reverse Transcriptase (Life Technologies). cDNA from blood samples was obtained using 750 ng RNA, with the same reaction conditions as above. For each sample a negative control was performed by omission of the reverse transcriptase (-RT control). Specific qPCR primers were designed using the online tool Primer-Blast provided by NCBI.

The primers sequences were as follows: for *GAPDH* 5′-CCTGCACCACCAACTGCTTA-3′ and 5′-TCTTCTGGGTGGCAGTGGATG-3′; for *ACTB* 5′-AGAGCTACGAGCTGCCTGAC-3′ and 5′-AGCACTGTGTTGGCGTACAG-3′; for *RPL19* 5′-CTAGTGTCCTCCGCTGTGG-3′ and 5′-AAGGTGTTTTTCCGGCATC-3′; *B2M* for 5′-AGATGAGTATGCCTGCCGTG-3′ and 5′-TCATCCAATCCAAATGCGGGC-3′; for *HBB* 5′-AGGAGAAGTCTGCCGTTACTG 3′ and 5′-CCGAGCACTTTCTTGCCATGA-3′; for *HBA1/2* 5′-TTCTGGTCCCCACAGACTCA-3′ and 5′-CAGGAACATCCTCTCCAGGG-3′; for *ALAS2* 5′-TGTCCGTCTGGTGTAGTAATGA-3′ and 5′-GCTCAAGCTCCACATGAAACT-3′; for *KDR* 5′-GACCGGCTGAAGCTAGGTAA-3′ and 5′-CGATGCTCACTGTGTGTTGC-3′; for *CD34* 5′-ACCACTAGCACTAGCCTTGC-3′ and 5′-GGCAGATGCCCTGAGTCAAT-3′.

Gene expression assays were performed using iQ™ SYBR® Green Supermix 2x (Bio-Rad Laboratories, Inc.), 400 nM final concentration of the corresponding forward and reverse primer (Sigma) and 10 ng/μL final concentration of cDNA samples, with CFX96 Touch™ Real-Time PCR Detection System (Bio-Rad Laboratories, Inc.).

### RT-qPCR data analysis

Differential expression of target genes was normalized to four reference genes (*GAPDH, ACTB, RPL19*, and *B2M)* expression.

Initially, the absolute expression value (C_T_) of a specific erythrocyte marker, *ALAS2*, was used to exclude blood contaminated samples and the relative expression ratio (fold change, FC) was calculated using the 2^−ΔΔC_T_^ method (Livak and Schmittgen, 2001).

Then, an additional normalization was performed using *ALAS2* expression values, to take into account the blood contamination of each brain tissue sample.

The “blood normalized” FC was calculated using the 2^−ΔΔC_T_^ method corrected for erythrocytes marker expression, as following:

$$\begin{array}{l} \;\Delta {\text{C}}_{\text{T }(“blood\;normalized”\;test)}=({\text{C}}_{\text{T}(target,\;test)}-{\text{C}}_{\text{T}(reference,\;test)})\\ \;-\;{\text{C}}_{\text{T}(erythrocyte\;marker,\;test).}\\ \Delta {\text{C}}_{\text{T}(“blood\;normalized”\;calibrator)}=({\text{C}}_{\text{T}(target,\;calibrator)}\\ \;-\;{\text{C}}_{\text{T}(reference,\;calibrator)})\\ \;-\;{\text{C}}_{\text{T}(erythrocyte\;marker,\;calibrator).}\\ \;\Delta \Delta {\text{C}}_{\text{T}(“blood\;normalized”)}=\Delta {\text{C}}_{\text{T}(“blood\;normalized”\;test)}\\ \;-\;\Delta {\text{C}}_{\text{T }(“blood\;normalized”\;calibrator).}\\ \;FC=2^{-\Delta \Delta {\text{C}}_{\text{T}}(“blood\;normalized”)}. \end{array}$$

ΔC_T_ were calculated subtracting the C_T_ of the housekeeping gene to the C_T_ of the target one, both for “test” (disease affected patient) and “calibrator” (control). Then, ΔΔC_T_ was calculated subtracting the average ΔC_T_ of age-matched control groups to the average ΔC_T_ of each disease groups.

To select age-matched control for each prion diseases and Alzheimer's disease group, the same sample size of non-neurodegenerative controls was chosen to match both median and average age of disease affected patients' group.

Fold change values smaller than 1 were converted using the equation-1/fold change, for ease of representation.

### Validation of “blood normalization” analysis method

In order to prove the robustness of the proposed method, we performed additional analysis as validation. We performed spiking experiments adding increasing concentrations of blood cDNA (from 0.1 pg to 1 ng, corresponding to four orders of magnitude) to a fixed amount of “contaminated” brain cDNA (10 ng, the same used in all our qPCR experiments). The validation experiments were carried out using blood samples from two healthy controls and two patients.

In these mixed samples, we analyzed the levels of *HBB, HBA1/2, ALAS2* and the four reference genes that we mentioned above.

### Statistical analysis

Test-F was used to verify the homoscedasticity of variance between ΔC_Ts_ of disease-affected and the related age-matched control group (α = 0.05). Level of significance was calculated using unpaired student *t*-test (two tails, *p* < 0.05) between ΔC_Ts_ of disease and control groups. Correlation was calculated using Pearson correlation equation (*R*).

### Double-labeling immunofluorescence and confocal microscopy

De-waxed sections, 4 microns thick, corresponding to cortical layer III, were stained with a saturated solution of Sudan black B (Merck, DE) for 15 min to block the autofluorescence of lipofuscin granules present in cell bodies, and then rinsed in 70% ethanol and washed in distilled water. The sections were incubated at 4°C overnight with mouse monoclonal anti-hemoglobin-α (1:50, Santa Cruz, TX, USA) or rabbit polyclonal anti-hemoglobin-β (1:50, Abcam, UK), and rabbit polyclonal antibody anti-GFAP (1:400, Diagnostic Biosystem, Barcelona, Spain) or mouse monoclonal anti-NeuN (1:100, Merck Millipore, MA, USA), respectively. After washing, the sections were incubated with Alexa488 (1:400, Molecular Probes, USA) fluorescence secondary antibodies. Nuclei were stained with DRAQ5™ (1:2,000, Biostatus, GB). After washing, the sections were mounted in Immuno-Fluore mounting medium (ICN Biomedicals, USA), sealed, and dried overnight. Sections were examined with a Leica TCS-SL confocal microscope.

### Data availability

All data generated and/or analyzed during the current study are available from the corresponding author on reasonable request.

## Results

### qPCR analysis

We obtained a list of 128 suitable samples, grouped as reported in Table 1. In general RNA integrity is a common issue when dealing with post-mortem tissue. Indeed, it is known to depend on several variables such as prolonged storage (Schoor et al., 2003), unrefrigerated post-mortem interval (Weis et al., 2007) and deterioration associated to brain acidosis during prolonged agony before death (Koppelkamm et al., 2011; Wang et al., 2012). After stringent RNA quality assessment, almost half of the collected samples displayed a high degree of degradation (RIN < 4) that did not allow their use in this study.

**Table 1:** Summary of sample groups used in the study.

**Group**	**Number (*n* =)**	**Mean age (ys ± SD)**
CTRL	30	–
sCJD	23	67 ± 13
gPrD	30	54 ± 12
vCJD	20	35 ± 13
iCJD	11	29 ± 5
AD	14	69 ± 10

*To select age-matched controls for each prion diseases and Alzheimer's disease groups, the same sample size of non-neurodegenerative controls was chosen to match both median and average age of disease affected patients' group*.

Complementary DNA was obtained by reverse transcription from the selected RNAs. Then, samples from patients and controls were analyzed by qPCR in parallel, to avoid bias due to samples handling.

It is known that normalization by an internal reference gene reduces tissue derived effects on RT-qPCR (Fleige and Pfaffl, 2006). The vast majority of RT-qPCR expression profiles found in the literature uses *GAPDH* (glyceraldehyde-3-phosphate dehydrogenase) as a reference gene for normalization (Penna et al., 2011). However, *ACTB* (actin-β), *B2M* (β_2_-microglobulin), and the ribosomal protein family seem to show good stability across different RIN-values (Koppelkamm et al., 2011) and brain samples (Penna et al., 2011). Therefore, we decided to use *GAPDH, ACTB, RPL19*, and *B2M* as references to normalize RT-qPCR data in order to consolidate the relative gene expression levels, given the high variability characterizing human samples.

**Figure 1:**
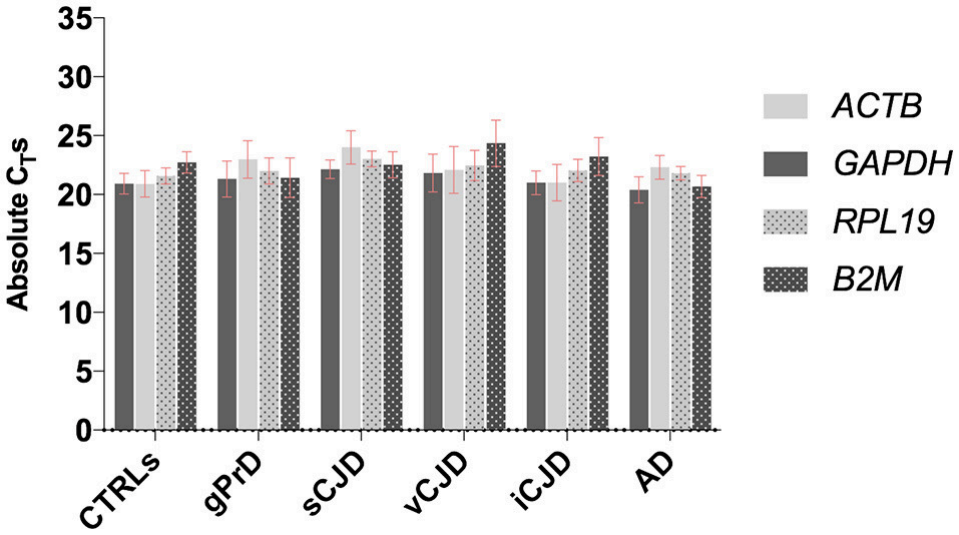
Comparison between *GAPDH, ACTB, RPL19*, and *B2M* stability across controls and patients. For each group, average values of absolute C_T_s among the samples are shown. Each sample was analyzed in triplicates.

First, the four housekeeping genes were analyzed across diseased and control cases in order to assess their expression stability (Figure 1).

As shown, all four genes selected for reference displayed comparable expression levels across all the analyzed groups, with very similar C_T_-values and standard deviation both within and across each group.

Hemoglobin is the major protein found in erythrocytes, and besides its high concentration at protein level, high amount of Hb transcripts were found in erythrocytes (Gotting and Nikinmaa, 2015). Therefore, we decided to perform a titration using serial dilution of blood tissue, comparing their Hb expression levels to one present in brain tissue, in order to understand how much the contaminating blood would affect our results related to brain tissue.

First, we observed that both *HBB* and *HBA1/2* expression was higher than reference genes in blood samples, highlighting the presence of a large amounts of Hb transcript within blood cells. Furthermore, we found that Hb transcripts are highly expressed even in very small amount of blood samples (C_T_ = 17 for *HBB* and C_T_ = 20 for *HBA1/2*) when compared to their expression levels in brain tissue (Figure S1).

In light of these results, dealing with material in which blood contamination is inevitable, we decided to analyze the expression levels of the erythrocyte marker, *ALAS2*, in order to account for the blood influence on the expression of our target genes.

**Figure 2:**
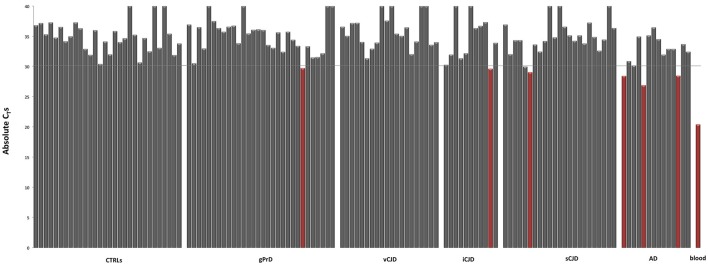
Blood erythrocyte markers expression across controls and patients. Absolute C_T_-values for *ALAS2* in control and neurodegenerative affected brain samples. “Blood contaminated” samples are shown in red. Pool of blood cDNA (*n* = 2) is used as positive control (in red).

The absolute quantification of *ALAS2* was previously used as threshold to exclude blood contaminated samples from gene expression analysis (Barbisin et al., 2014). However, the vast majority of our samples presented small blood traces as we inferred from C_T_s ≤ 35 for *ALAS2* (Figure 2). Thus, we decided to exclude samples showing C_T_ ≤ 30 for *ALAS2*, considering them as the ones with blood contamination, which could sensibly influence the reliability of results, related to the regulation of both hemoglobin chains.

The mRNA levels of the selected human frontal cortex samples were calculated using the 2^−ΔΔC_T_^ method. No significant dysregulation among the different diseases patient's groups, when compared to age-matched controls, was evident. In particular, FC ≤ 2 were observed for both *HBB* and *HBA1/2* after normalization vs. all four housekeeping genes, with opposite trend between the two chains in most of groups, making difficult to obtain reliable conclusions (data not shown).

**Figure 3:**
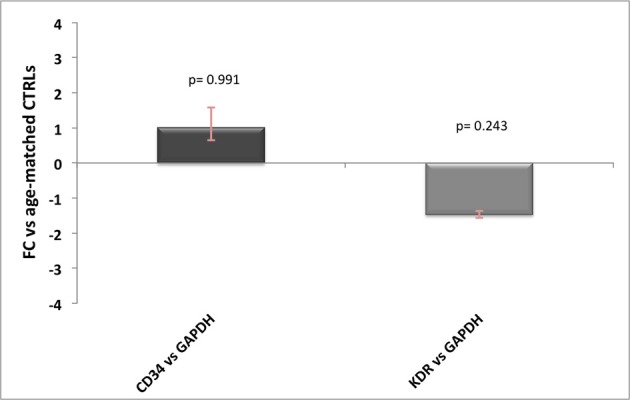
Endothelial markers expression levels in AD patients. Relative expression levels of *CD34* and *KDR* normalized against *GAPDH* in AD patients vs. age-matched controls, with related *p*-values. For FC-values smaller than 1 the reciprocal (−1/FC) is reported, for ease of representation.

One other source of contamination of Hb transcripts would be the endothelium, especially for *HBA1/2* gene, which has been previously reported to be well expressed in endothelial cells. Since neither AD nor prion diseases are known for alterations in the number of vessels, the influence of endothelial Hb should be similar across controls and patients. However, to better address this point we tested seven commonly known endothelial and/or angiogenesis markers (*ICAM1, vWF, PECAM1, KDR, CD34, TEK*, and *CDH5*) on brain cDNA (10 ng) and blood cDNA (10 ng) and found out that only two of them were not detectable in blood (data not shown). Then, we performed qPCR analysis for *KDR* and *CD34* (Hirakawa et al., 2003) in AD and control samples and we did not detect any significant difference between the groups (Figure 3). Therefore, even if we are not able to quantify the precise influence of endothelial cells on our results, we are pretty confident that this would affect equally controls and diseased samples without impairing our final results.

### Blood-normalized gene expression analysis

The high hemoglobin genes expression we observed in blood (Figure S1), in line with previous findings (Gotting and Nikinmaa, 2015), suggested us that even minute blood traces could influence brain hemoglobin gene expression. Therefore, we wondered if the inconclusive results obtained performing the 2^−ΔΔC_T_^ analyses might have been jeopardized by the blood traces not taken in consideration by the *ALAS2* C_T_ cutoff value (C_T_ ≤ 30) used to exclude blood-contaminated samples.

We also show that besides hemoglobin, reference genes are highly expressed in very small amounts of blood (Figure S1), suggesting that hemoglobin genes expression levels in brain could be influenced also by the different expression of housekeeping genes among tissues.

**Figure 4:**
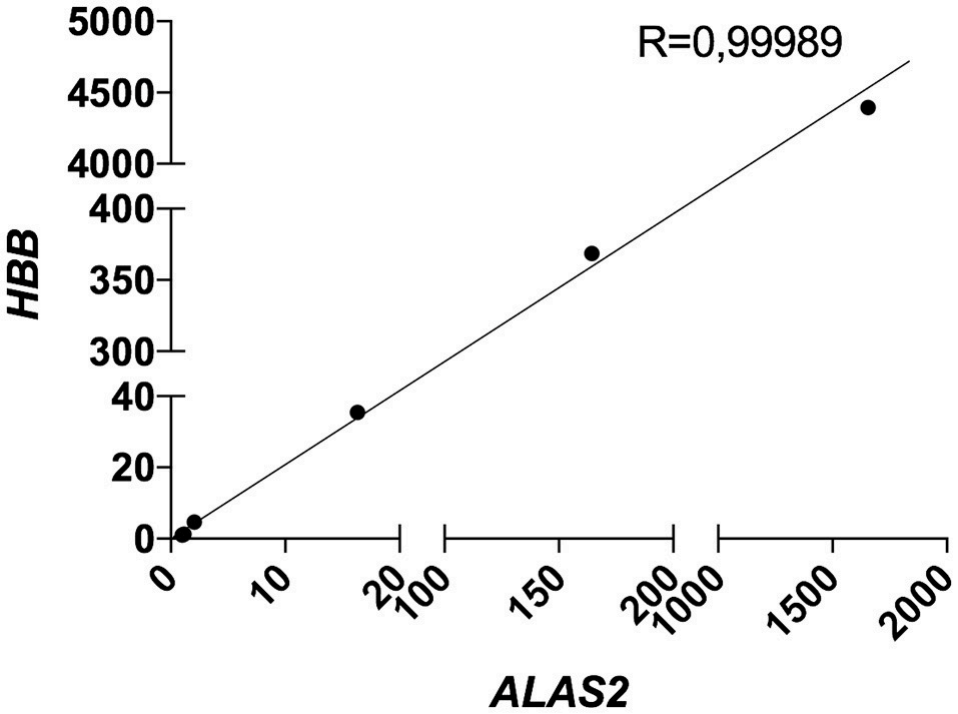
Correlation between *HBB* and *ALAS2* expression in brain samples (pool of 2 AD samples) containing serially diluted amounts of blood cDNA (pool of 2 healthy controls and 2 patients). R, Pearson correlation coefficient.

**Figure 5:**
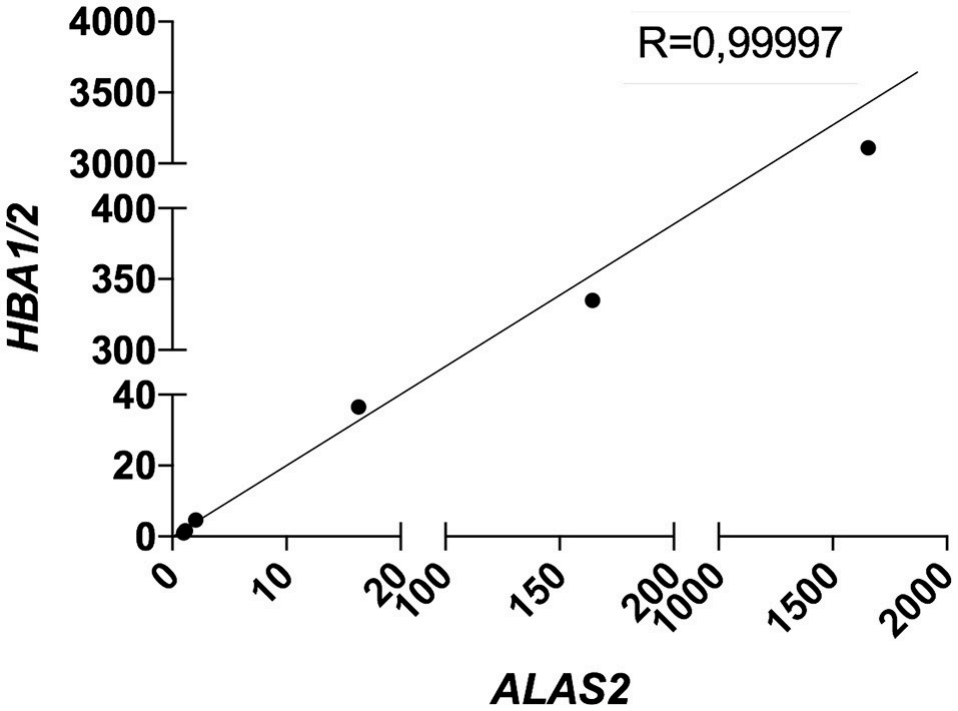
Correlation between *HBA1/2* and *ALAS2* expression in brain samples (pool of 2 AD samples) containing serially diluted amounts of blood cDNA (pool of 2 healthy controls and 2 patients). R, Pearson correlation coefficient.

Therefore, we decided to include the expression level (C_T_) of *ALAS2* in ΔC_T_ formula in order to normalize the gene expression of both targets and housekeeping genes for each single sample on its specific blood content. Furthermore, we decided to re-introduce the previously excluded blood contaminated samples. In order to validate the robustness and efficacy of our correction method, we performed several spiking experiments adding different amounts of blood cDNA to the same amount of brain cDNA. As showed, *ALAS2* expression is decreasing with the reduction of blood added to the brain, in the very same manner as *HBB* and *HBA1/2*, confirming its specificity as blood marker (Figures S2, S3). We analyzed the correlation between the relative expression of *ALAS2* and *HBB* (Figure 4) or *HBA1/2* (Figure 5), and the resulting *r*-values were 0.99989 and 0.99997, respectively.

Importantly, when we then analyzed the same samples with our blood-normalized method, the expression levels of both *HBB* and *HBA1/2* of all samples containing increasing amounts of blood were comparable to the one of the brain alone (Figures S2, S3), with FC lying between 1 and 2, where FC ≥ 2 is commonly used as the threshold for defining an upregulation, especially for highly heterogeneous samples as human ones. We obtained similar results normalizing the data against *ACTB, RPL19*, and *B2M* (data not shown).

**Figure 6:**
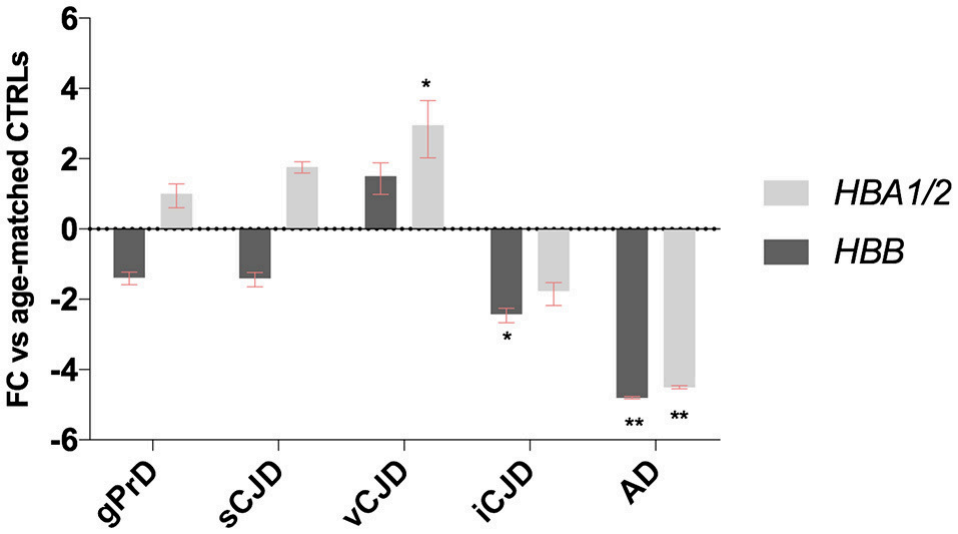
Blood-normalized *HBB* and *HBA1/2* expression. Relative expression levels of *HBB* and *HBA1/2* against *GAPDH* and against *ALAS2* in gPrD, sCJD, vCJD, iCJD, and AD patients. ^*^p < 0.05, ^**^p < 0.005.

This modified RT-qPCR data analysis method revealed significant dysregulation of both hemoglobin gene chains in some groups, with very similar results normalizing targets to all four reference genes (Figure 6, Figures S4–S6).

While in genetic prion diseases and sporadic CJD groups *HBB* and *HBA1/2* expression levels did not show any dysregulation, in AD patients both hemoglobin transcripts were strongly down-regulated. Given that in literature is well known that gender plays an important role in AD occurrence, the control group was both age- and sex-matched (Figure S7 and Table S1), even though we did not observe any age- or sex-related variability neither in *HBB* nor in *HBA1/2* (Figures S8, S9).

Concerning acquired prion diseases, iCJD patients exhibited a down-regulation of both chains as well, even if statistical significance was reached only for *HBB*. On the contrary, vCJD patients showed an up-regulation of Hb transcripts, which was significant for *HBA1/2* (Figure 6).

Similar results were obtained normalizing data against all the other three reference genes (Figures S4–S6).

### Immunofluorescence and confocal microscopy analysis

**Figure 7:**
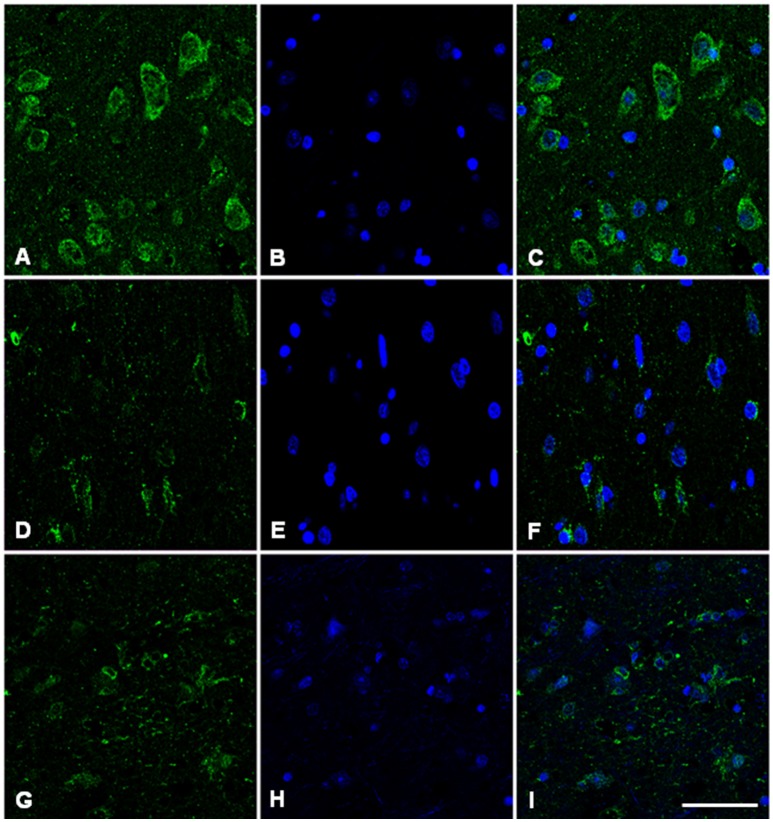
Hemoglobin beta-chain in the frontal cortex of control subject **(A–C)**, Alzheimer's disease **(D–F)** and sCJD **(G–I)**. Immunofluorescence and confocal microscopy for hemoglobin B chain **(A,D,G)** and nuclei **(B,E,H)** showing reduced hemoglobin immunoreactivity in neurons of AD and sCJD (F, I merge) compared to control (C merge). Nuclei stained with DRAQ5™. Bar = 50 μm.

**Figure 8:**
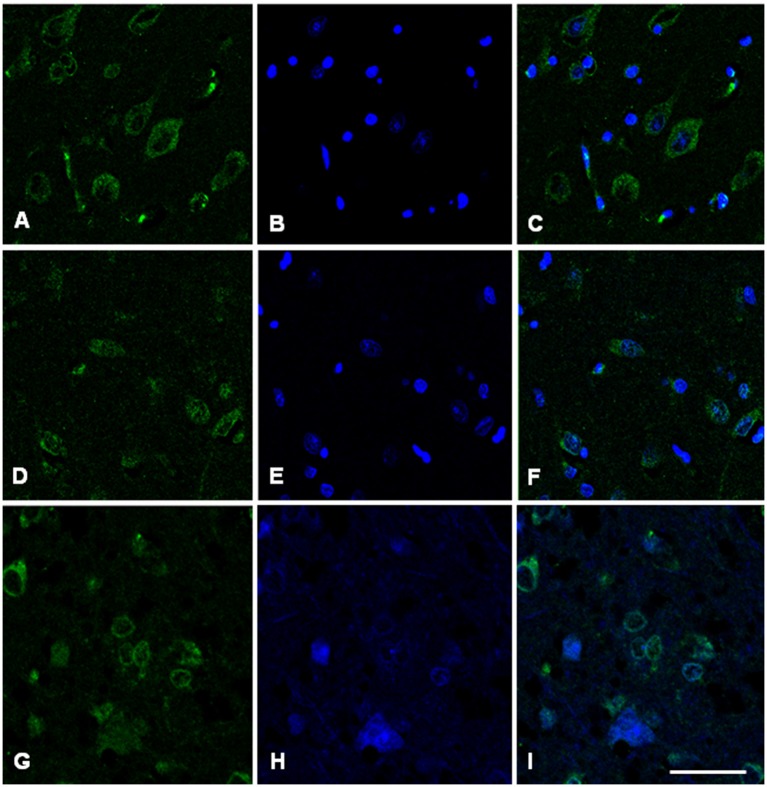
Hemoglobin alpha-chain in the frontal cortex of control subject **(A–C)**, Alzheimer's disease **(D–F)** and sCJD **(G–I)**. Immunofluorescence and confocal microscopy for hemoglobin α-chain **(A,D,G)** and nuclei **(B,E,H)** showing hemoglobin immunoreactivity in neurons of AD patients (F, I merge) compared to control (C, merge). Nuclei stained with DRAQ5™. Bar = 50 μm.

Immunofluorescence and confocal microscopy analysis was performed on AD and sCJD frontal cortex slices. We observed that both Hb β and Hb α immunoreactivity is reduced in neurons in AD when compared to control samples, recapitulating the results observed at the transcriptional level (Figures 7, 8). Concerning sCJD slices, we observed a slight decrease of Hb β immunoreactivity in neurons, mirroring the trend of the mRNA expression (Figure 7). In contrast, Hb α neuronal immunoreactivity was preserved or increased when compared to controls (Figure 8). Hemoglobin immunoreactivity was barely observed in astroglia, excepting one single case in which immunoreactivity for hemoglobin α-chain, but not β-chain, was evident in astrocytes (Figures S10, S11).

## Discussion

In recent years, hemoglobin genes and protein dysregulation has been associated with several neurodegenerative diseases in humans, including AD, PD, MSA, MS (Broadwater et al., 2011; Ferrer et al., 2011; Shephard et al., 2014; Brown et al., 2016; Mills et al., 2016), and with prion disease in mouse (Booth et al., 2004a), macaque (Barbisin et al., 2014), and cattle models (Xerxa et al., 2016).

To better understand the hemoglobin genes involvement in human prion disorders, we investigated their expression levels across different prion diseases, either sporadic acquired or genetic.

In mammalian enucleated erythrocytes, the α and β chains forming hemoglobin are present at high concentrations, resulting in an efficient O_2_ transport mechanism (Schechter, 2008). However, Hb chains have been detected also in a plethora of non-erythroid cells including neurons and glial cells (Biagioli et al., 2009).

As a first step, based on active transcription within human red blood cells (Kabanova et al., 2009) and, more importantly, given that Hb transcripts are present at high levels in mammalian mature circulating erythrocytes (Gotting and Nikinmaa, 2015), we hypothesized that the presence of blood in human frontal cortex could potentially jeopardize hemoglobin gene expression dysregulation. In addition, Wu and colleagues suggested that cerebral blood supply could represent another source of brain hemoglobin. For example, the commonly observed AD cerebrovascular lesions induce transient infiltration of erythrocytes from circulation into the brain parenchyma, thus influencing hemoglobin levels (Wu et al., 2004).

Only few studies concerning Hb dysregulation in neurodegenerative-affected patients have considered red blood cells (RBCs) hemoglobin contribution. Tissue contamination with RBCs could influence the neuronal/glial hemoglobin expression analysis. Ferrer and colleagues reported neuronal hemoglobin to be reduced in AD and other neurodegeneration affected brains using both immunohistochemistry and immunofluorescence. Due to intravascular RBCs contamination, they considered Western blot technique not useful to analyze minor modifications of hemoglobin in nervous system (Ferrer et al., 2011).

To confirm this idea, we analyzed all our samples and we observed that even small amount of blood-derived RNA showed high levels of both Hb and reference genes. Thus, we decided to include the C_T_-value of *ALAS2* in ΔC_T_ formula, for both patients and control samples. In particular, we subtracted the C_T_ value of *ALAS2* to the canonical ΔC_T_, normalizing the expression of both hemoglobin (*HBB* and *HBA1/2*) and housekeeping genes (*GAPDH, ACTB, RPL19, B2M*) for erythrocytes content. The resulting “blood normalized” ΔΔC_T_ was used to calculate the fold change.

Re-analyzing RT-qPCR data with this improved method, we observed a strong down-regulation of both hemoglobin gene transcripts in patients with Alzheimer's disease. Similarly, we observed reduced hemoglobin protein levels with immunofluorescence staining, further corroborating the robustness of our analysis.

Regarding prion diseases groups, dysregulated *HBB* and *HBA1/2* transcript levels followed different trends depending on the disease type. Genetic and sporadic prion diseases affected patients did not exhibit any alteration for what concerns hemoglobin genes expression, suggesting that prion infection may exert a limited impact on the transcriptional machinery involved in neuronal hemoglobin expression.

However, the analysis of *HBB* and *HBA1/2* mRNA levels in acquired prion diseases patients revealed some differences, suggesting that exogenous prions might exert an impact on Hb metabolism. In particular, while an up-regulation for both hemoglobin genes was evident in vCJD patients (statistically significant for *HBA1/2* expression), iCJD group exhibited a down-regulated Hb gene expression (statistically significant for *HBB* expression).

One explanation for the different expression levels reported between the two Hb chains could depend on primers pair designed for *HBA1/2* transcripts, which bind both the human α-2 (*HBA2*) and α-1 (*HBA1*) coding sequences. Therefore, being primers pair for α-globin genes able to recognize two different transcripts, the signal contribution given by qPCR amplification might be different between *HBA1/2* and *HBB*. Considering also the fact that *HBA1* and *HBA2* could be differently regulated, all these observations might explain the lack of correlation seen in some prion groups in the expression trend between the two hemoglobin chains genes. Furthermore, although it has been reported that α and β subunits function as a tetramer both in erythrocytes and neurons (Russo et al., 2013), they may also act independently one from another. Indeed, while Hb α regulates NO release in vascular endothelial cells independently of Hb β (Straub et al., 2012), tetramer of β-globin chains (HbH) were shown to possess a higher binding affinity for O_2_ than classical α_2_β_2_ hetero-tetramers (Bellelli et al., 2006). The different levels of *HBB* and *HBA1/2* dysregulation observed in some diseases groups could be explained also by the independent functions adopted by the two hemoglobin chains in brain tissue or even in different cell populations, given that we analyzed RNA from whole frontal cortex without distinguishing white from gray matter, where differential expression of Hb genes has been reported (Mills et al., 2015).

Nevertheless, our results suggest that brain hemoglobin dysregulation is different among patients suffering from various prion diseases, supporting a prion strain-specific on hemoglobin gene expression.

In particular, this finding suggest that hemoglobin genes could be used as TSEs strain determinants (Booth et al., 2004b) or may contribute to strain-specific neurodegenerative processes (Skinner et al., 2006).

Indeed, PrP^Sc^ to PrP^C^ binding is necessary to produce new misfolded isoforms, therefore it is plausible that different PrP^Sc^ strains could activate specific PrP^C^ molecular scaffolding partners. In this way, a variety of signaling pathways could be switched on/off, leading to the transcriptional inhibition or activation of many genes, among which hemoglobin coding ones.

In this view, it seems that BSE prion strain (as in vCJD group) is responsible for an up-regulation of hemoglobin genes. Our findings seem to be in contrast with previous results in BSE-infected macaques, where both *HBB* and *HBA1/2* were strongly down-regulated.

However, this discrepancy could be explained by the fact that, even if the gene expression analysis in macaques brain was performed using *ALAS2* C_T_ to exclude any major effect of potential blood contamination, RT-qPCR data analysis were not assessed using the “blood normalized” 2^−ΔΔC_T_^ method. Our findings imply that even subtle presence of blood hemoglobin transcripts, not excluded using a C_T_ cutoff value for *ALAS2*, could influence brain hemoglobin gene expression. This suggests that small blood traces may have probably influenced Hb genes expression analysis in macaque brains. Another source of hemoglobin transcript contamination could be endothelial cells where *HBA1/2* expression has been reported (Straub et al., 2012). However, neither AD nor prion diseases are known for alterations in the number of vessels in comparison to age-matched healthy subjects, therefore the influence of endothelial Hb should be similar across controls and patients. Despite this, we investigated the expression levels of two commonly known specific endothelial genes, *CD34* and *KDR* (*Vascular Endothelial Growth Factor Receptor 2*) and we did not observe any significant difference between controls and patients (Figure 3).

Moreover, opposite results concerning hemoglobin expression between vCJD patients and intracranially BSE-infected macaques may depend on the different routes through which they have been exposed to BSE. Indeed, the single macaque which was orally challenged with BSE (B6) showed a marked up-regulation of *HBB* transcript, reinforcing this hypothesis. We can hypothesize that a combination of strain and route of infection could be responsible for specific hemoglobin genes alterations. In particular, brain-directed BSE inoculation in macaques could produce metabolic insults resulting in a reduction of hemoglobin transcription, in a similar way through which aggregating protein deposits are responsible for a reduced neuronal hemoglobin expression (Ferrer et al., 2011).

It remains to clarify the molecular events through which oral BSE prion strain consumption elicits the up-regulation of hemoglobin gene (statistical significant for *HBA1/2*) in CNS of vCJD patients. One possible explanation could come from some neuronal hemoglobin behavioral effects that were recently shown overexpressing both α- and β-globin chains in mice *substantia nigra*. Indeed, upon Hb overexpression, mice presented motor skill learning impairments (Codrich et al., 2017), suggesting a possible role of Hb in neuronal damage occurring in *substantia nigra* of PD affected patients. In a similar way, the impaired coordination commonly observed in vCJD cases (Spencer et al., 2002), could derive from the aggregation of Hb, probably originating from its transcriptional up-regulated levels.

Furthermore, the Hb ability to bind to Aβ, enhancing its aggregation (Wu et al., 2004; Chuang et al., 2012), suggests that a similar interaction could be hypothesized between hemoglobin and BSE prion strain. In particular, up-regulated Hb levels could increase the aggregation of this peculiar prion strain, facilitating the development of vCJD.

One other cause responsible for the lack of clarity concerning hemoglobin genes dysregulation in some patients groups could originate from the mixed cell populations present in frontal cortex tissue from which RNA was extracted. This hypothesis is corroborated by previous findings which showed up-regulated hemoglobin genes (*HBB, HBA1, HBA2*) expression when white matter was compared with gray matter of MSA patients (Mills et al., 2015).

We can hypothesize that in AD, and probably also in iCJD and sCJD, when down-regulated Hb is not available for this function, cells get damaged by the defective oxygen homeostasis (Barbisin et al., 2014).

Since hemoglobin is the most abundant source of peripheral iron in humans, one clear implication of its dysregulation would be iron homeostasis alteration.

Iron metabolism alterations were observed in the central nervous system of many neurodegenerative disease affected patients. The decrease in Hb expression found in AD, iCJD, and sCJD could determine an increased amount of free iron which is highly toxic due to the generation of reactive oxygen species via the Fenton and Haber-Weiss reactions (Singh et al., 2014).

For prion diseases, the released iron would be bound by ferritin, creating a redox-active and cytotoxic complex with PrP^Sc^, ultimately lead to iron imbalance (deficiency phenotype) and to an increase of PrP^Sc^ toxic species (Singh et al., 2014) supporting the progression of the disease.

Even though only AD and sCJD cases were available for immunofluorescence and confocal microscopy, in general this analysis confirms the results observed at the transcriptional level. As already mentioned, Hb α and Hb β are probably regulated in different ways. While in AD samples both proteins and transcripts are consistently down-regulated, sCJD cases are more intriguing. Indeed, while Hb β is barely detectable, Hb α is still present in many cells. Therefore it could be that protein expression does not exactly reflect transcription in sCJD. However, in a single case of sCJD we observed a great Hb α immunoreactivity in astrocytes (Figures S10, S11), supporting the hypothesis of a different regulation and/or expression of both chains in different cell types, particularly neurons and astrocytes. This may also explain the lack of significant dysregulation of Hb transcripts in sCJD samples, given that the qPCR results come from mixed cell populations.

Our results suggest that brain hemoglobin could be an attractive candidate marker of neurodegeneration. Dysregulated levels of a protein with putative roles in O_2_ brain homeostasis and balance of redox system could be a sign of functional alterations occurring in brain of patients affected by neurodegenerative diseases. Moreover, through gene expression profiling is possible to identify specific molecular targets able to discriminate among different infectious agents (Booth et al., 2004a). In this context, marked differences in hemoglobin genes expression between sCJD and AD patients could represent a specific response of affected brain tissue to different neurodegeneration causing proteins deposits. In particular, our findings suggest that Aβ is able to activate molecular response pathway, which ultimately leads to a strong down-regulation of both hemoglobin gene expression.

## Conclusions

Hemoglobin gene expression alterations occurring in neurodegeneration-affected brains are not well documented and their role in disease remains elusive. In our gene expression analysis we propose a correction method for the presence of blood in tissue samples. These results strongly suggest that the neurodegenerative-specific hemoglobin mRNA level dysregulations can be associated to neuronal/glial populations. Future studies may discriminate in which cell types these dysregulations occur.

We can infer that only the BSE prion strain acquired via a peripheral route, as in vCJD patients, is able to cause an up-regulation of *HBA1/2* gene expression, while the various strains associated with hGH-iatrogenic CJD cases seem to be responsible for down-regulation of *HBB* gene expression. Other prion strains causing human genetic TSEs, sCJD strains, seem not to be responsible for any major Hb gene dysregulation. These findings suggest a strain-specific involvement of Hb genes in response to prion accumulation in affected brains.

Given the diverse tropism characterizing different prion strains, additional Hb gene expression analysis should be performed to understand how other brain regions are affected by hemoglobin dysregulation.

These alterations occur as either early or late consequence of the disease and may represent a susceptibility factor that influences the onset of the pathology.

Most importantly, a significant down-regulation of both *HBB* and *HBA1/2* is evident in AD patients. Given the high number of clinical phenotypic similarities and comparable age of disease onset between sCJD and AD patients, the difference in Hb gene expression open the possibility to develop a diagnostic test in order to perform differential diagnosis. Validation of these results in more readily accessible tissues, such as CSF or nasal brushing samples, could be helpful for clinicians in the correct interpretation of neurodegenerative symptoms.

Even though a clear correlation between hemoglobin gene expression and neurodegeneration has not yet been defined, taken together our findings strongly suggest a brain hemoglobin involvement in neurodegenerative processes.

## Author contributions

SV and GL conceived and designed the study. SV, FM, MZ, GG, FT, SH, J-PD, GZ, JI, MC, IF, GK, and GL contributed to the acquisition of samples and analysis of data. SV, MZ, MC, IF, and GL contributed in drafting a significant portion of the manuscript or figures. All authors approved the final version of the manuscript.

### Conflict of interest statement

The authors declare that the research was conducted in the absence of any commercial or financial relationships that could be construed as a potential conflict of interest.
