# Supplementary material for: Hemoglobin mRNA Changes in the Frontal Cortex of Patients with Neurodegenerative Diseases
Source: Front Neurosci. 2018 Jan 22;12:8. doi: 10.3389/fnins.2018.00008 (PMC5786544; doi:10.3389/fnins.2018.00008)
Supplement: Supplementary file 1 [file DataSheet1.pdf]

# Supplementary material for

# "Hemoglobin mRNA changes in the frontal cortex of patients with neurodegenerative diseases"

Vanni S[1], Zattoni M[1], Moda F[2], Giaccone G[2], Tagliavini F[2], Haïk S[3], Deslys JP[4], Zanusso G[5], Ironside JW[6], Carmona M[7], Ferrer I[8], Kovacs GG[9] and Legname G[1*]

**Figure S1. Titration of reference and hemoglobin genes expression levels in blood samples.** Absolute $C_T$ for target (*HBB* and *HBA1/2*) and two reference genes (*ACTB* and *RPL19*) of blood and brain samples are shown. Pool of blood and brain cDNA samples (n = 2 each) were prepared.

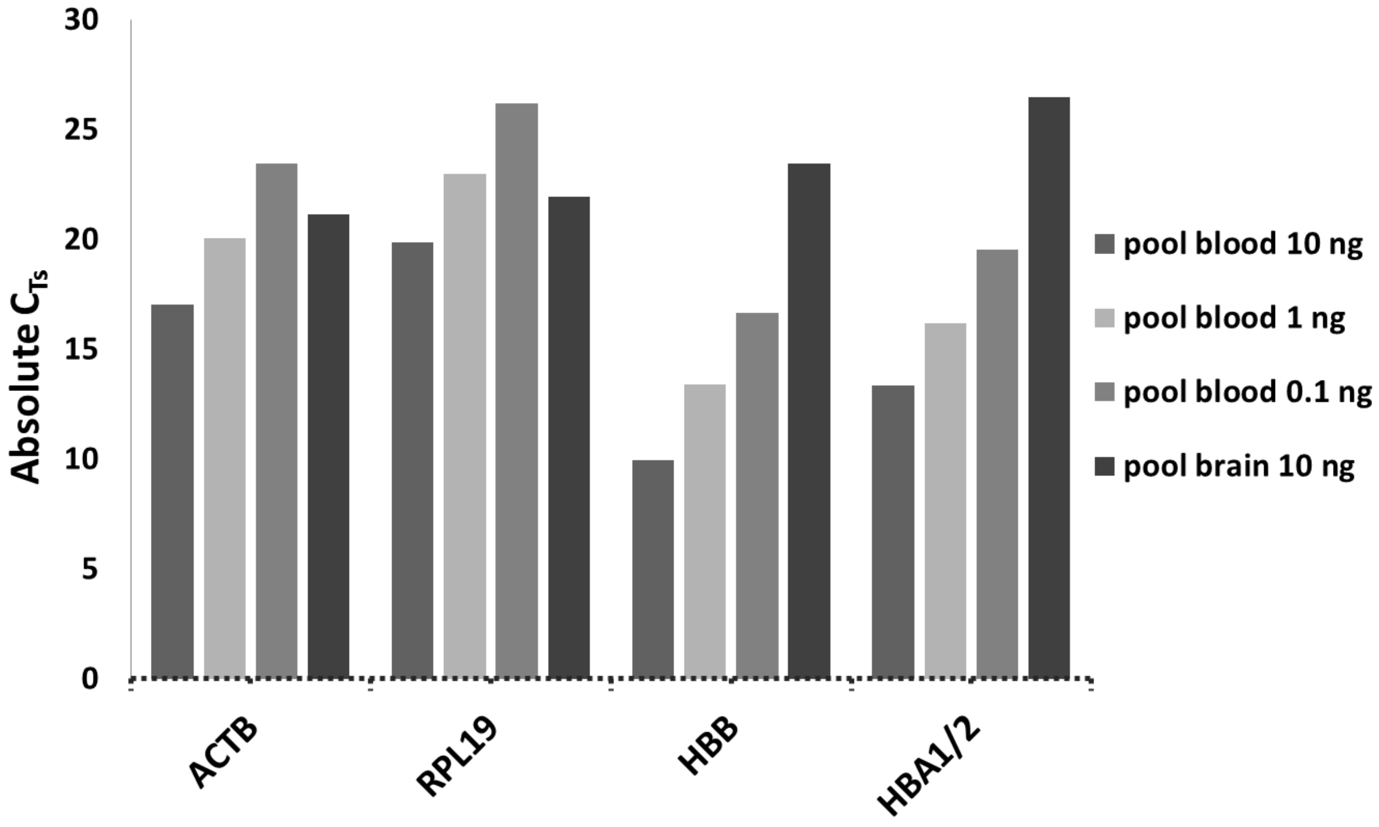

**Figure S2. qPCR validation for the "blood normalization" method with blood cDNA from healthy controls.** Relative expression levels of *HBB* and *HBA1/2* against *GAPDH* with and without *ALAS2* normalization in brain samples (pool of 2 AD samples) containing serially diluted amounts of blood cDNA (pool of 2 healthy controls).

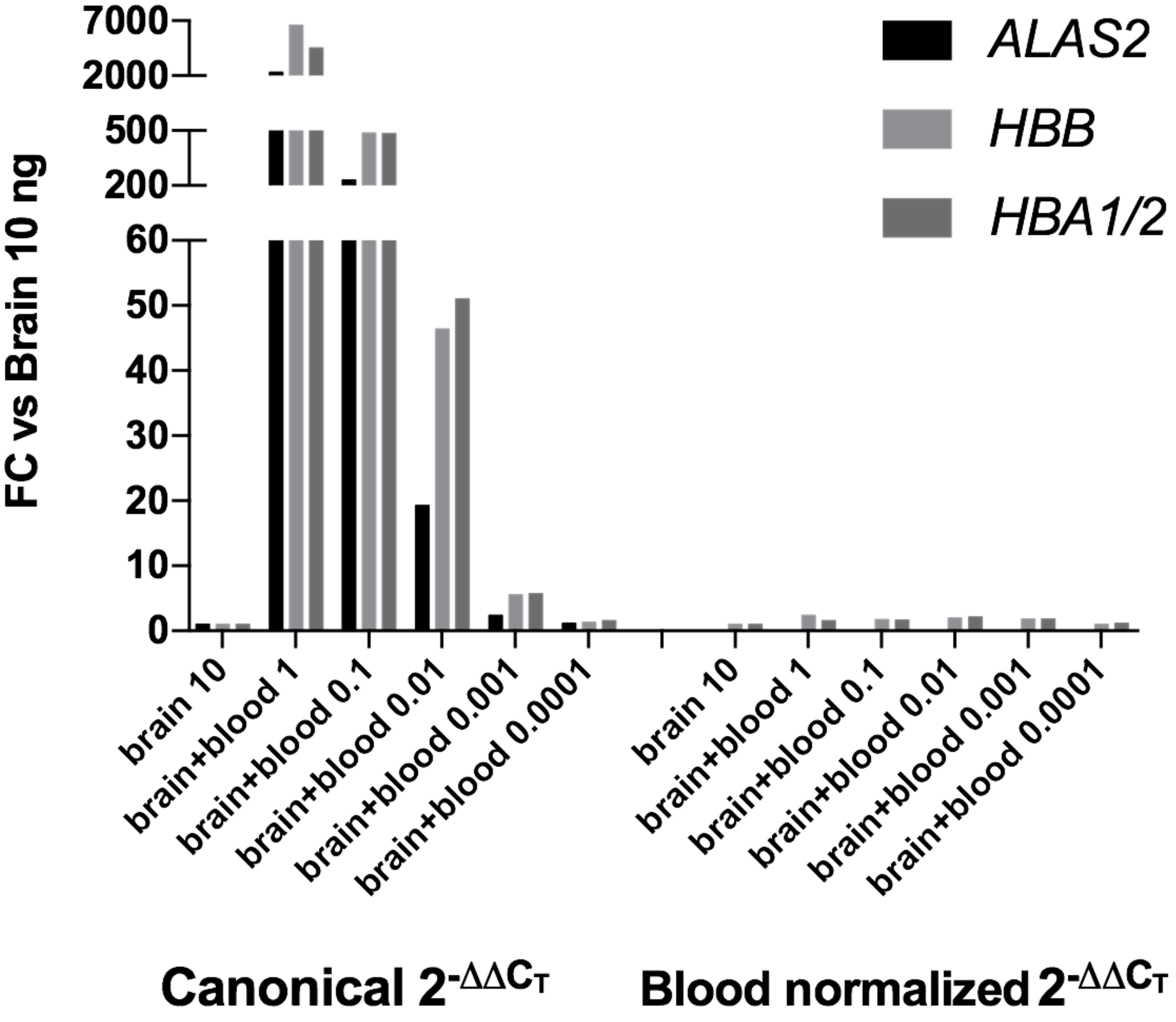

**Figure S3. qPCR validation for the "blood normalization" method with blood cDNA from diseased patients.** Relative expression levels of *HBB* and *HBA1/2* against *GAPDH* with and without *ALAS2* normalization in brain samples (pool of 2 AD samples) containing serially diluted amounts of blood cDNA (pool of 2 patients).

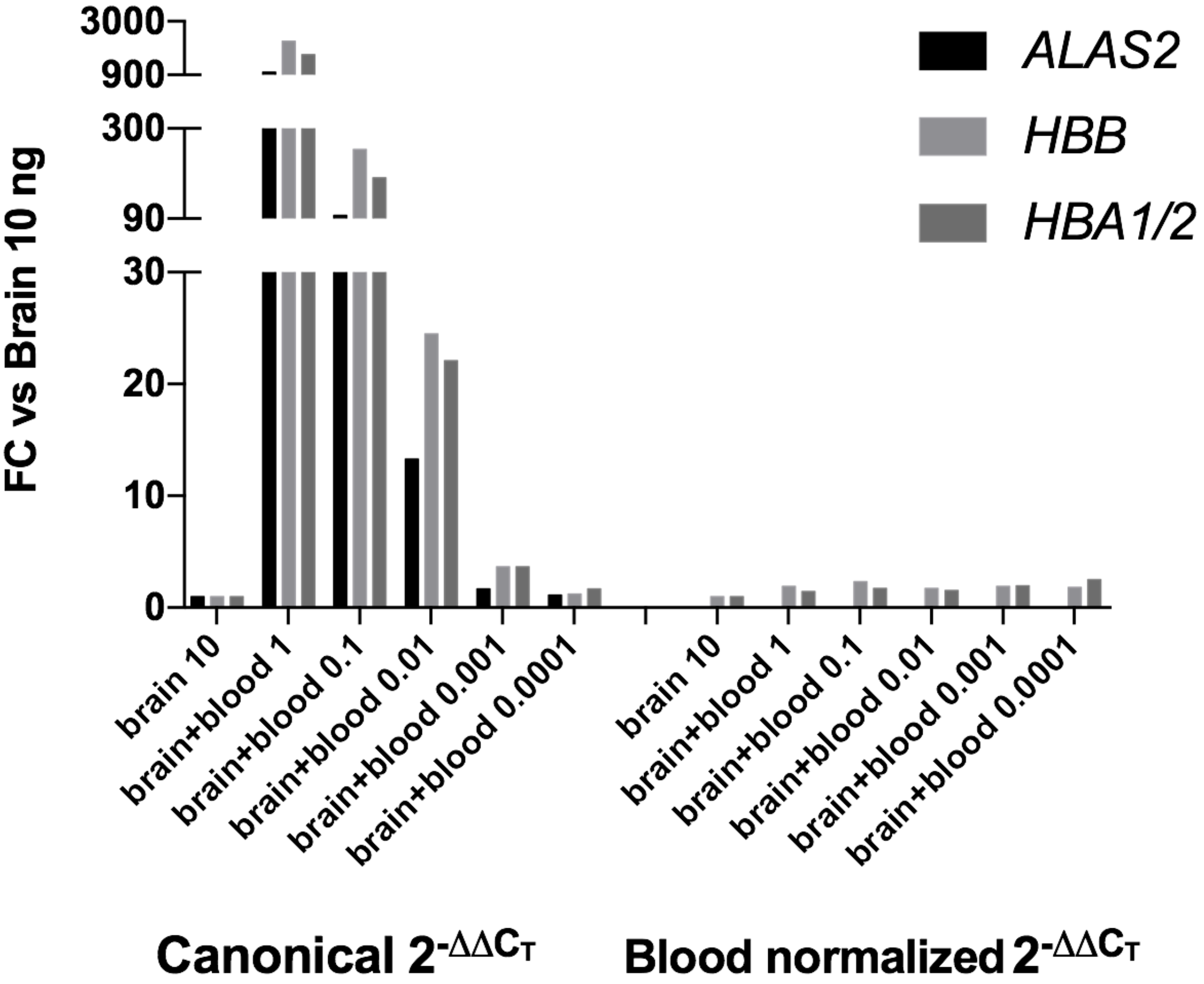

**Figure S4. Blood-normalized *HBB* and *HBA1/2* expression.** Relative expression levels of *HBB* and *HBA1/2* against *ACTB* and against *ALAS2* in gPrD, sCJD, vCJD, iCJD and AD patients. *=p<0.05, **=p<0.005

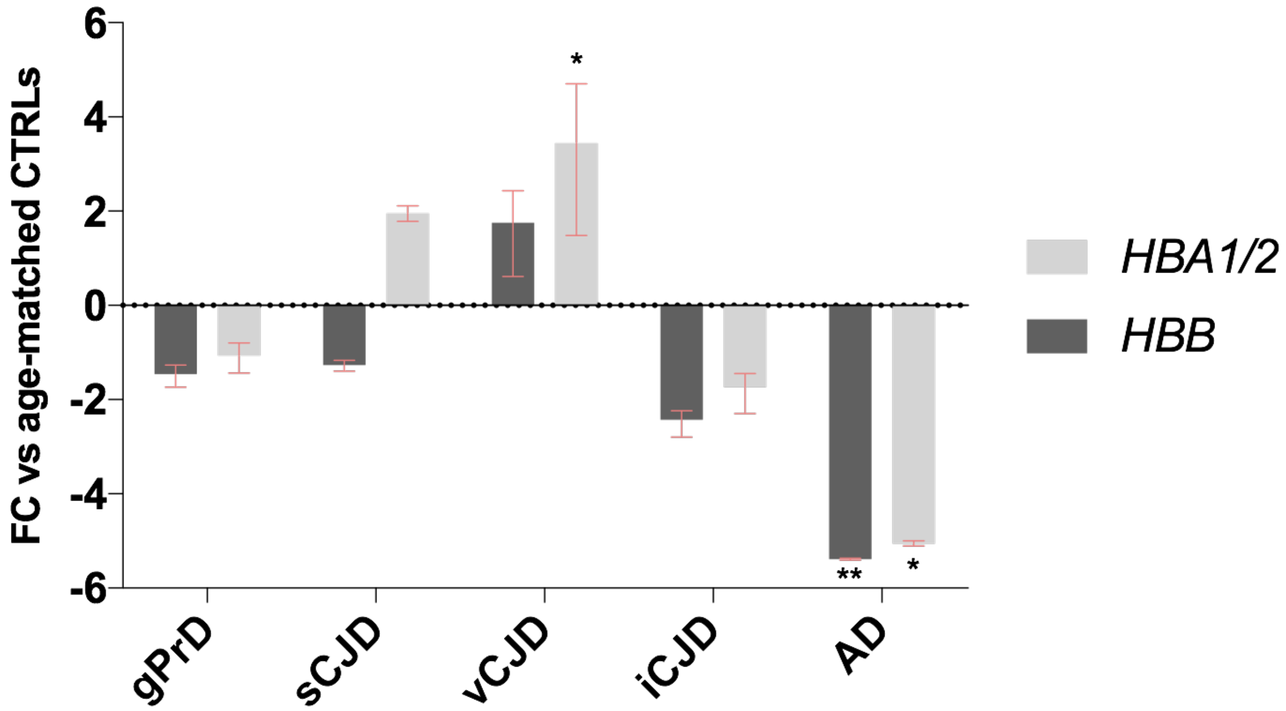

**Figure S5. Blood-normalized *HBB* and *HBA1/2* expression.** Relative expression levels of *HBB* and *HBA1/2* against *RPL19* and against *ALAS2* in gPrD, sCJD, vCJD, iCJD and AD patients. *=p<0.05

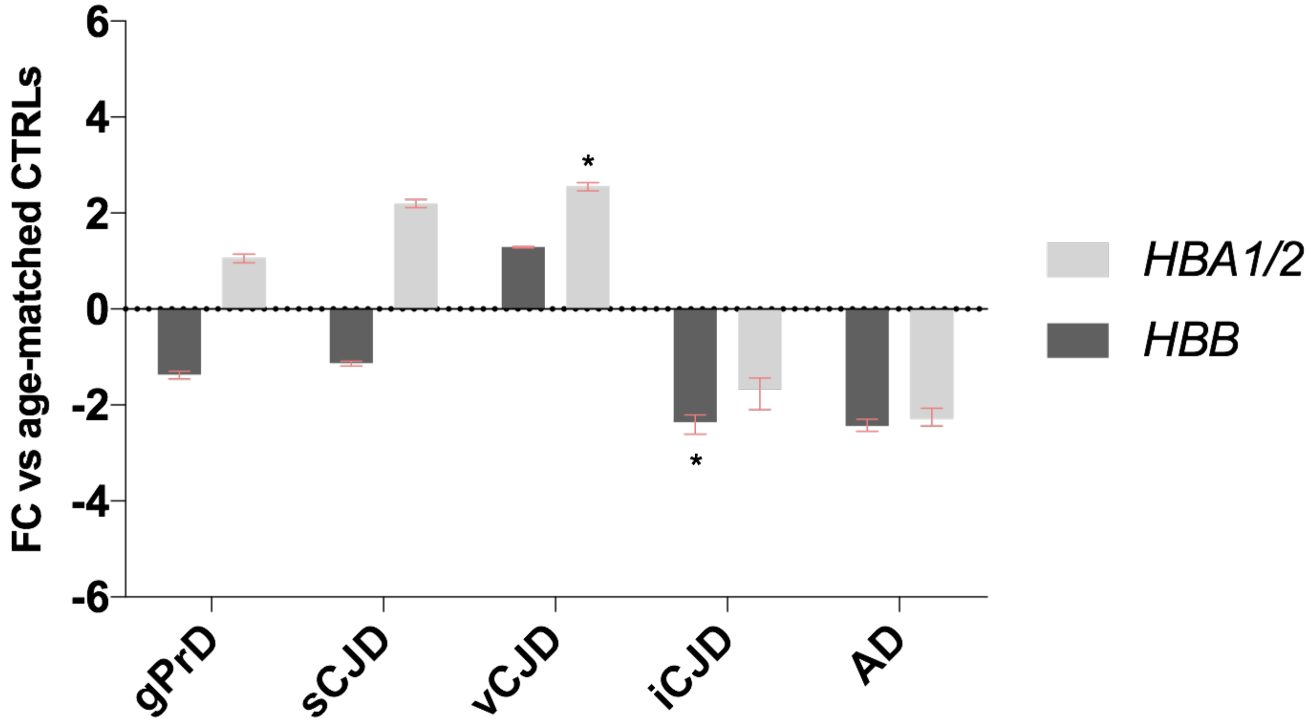

**Figure S6. Blood-normalized *HBB* and *HBA1/2* expression.** Relative expression levels of *HBB* and *HBA1/2* against *B2M* and against *ALAS2* in gPrD, sCJD, vCJD, iCJD and AD patients. *=p<0.05, **=p<0.01

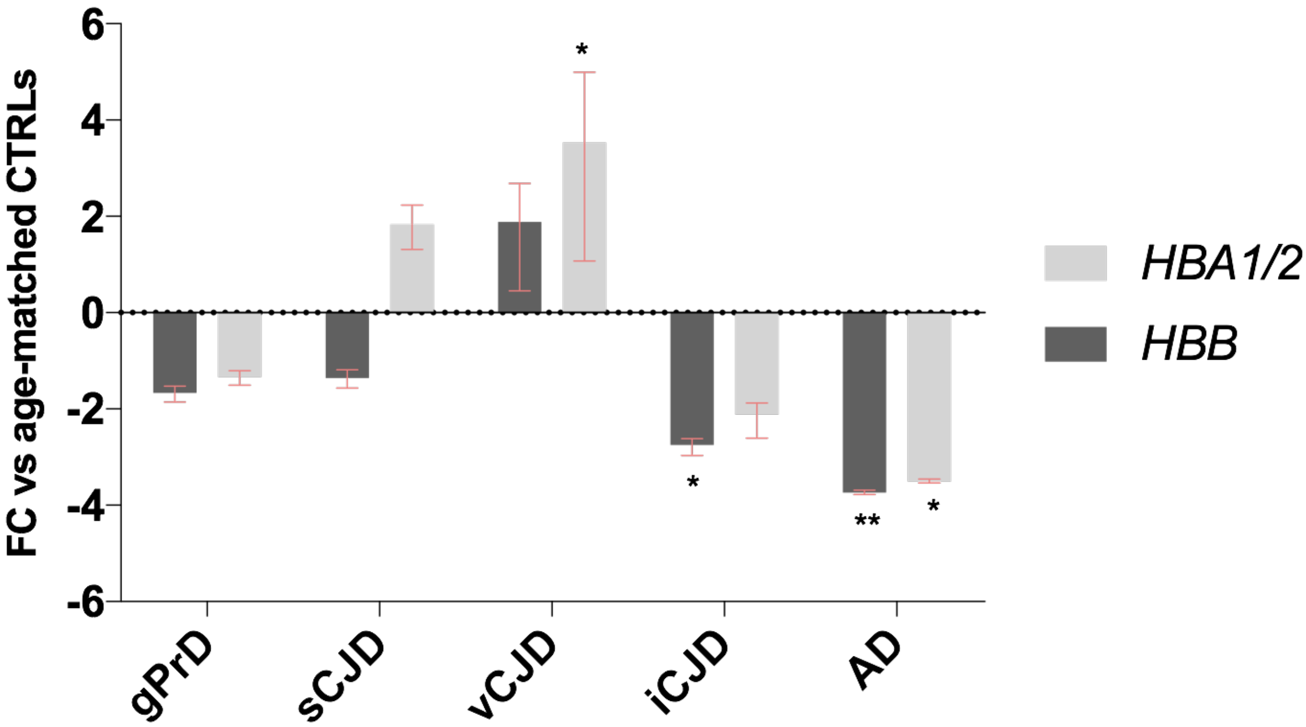

**Figure S7. Comparison of mean age between AD group and related healthy controls.** *n.s,* not significant

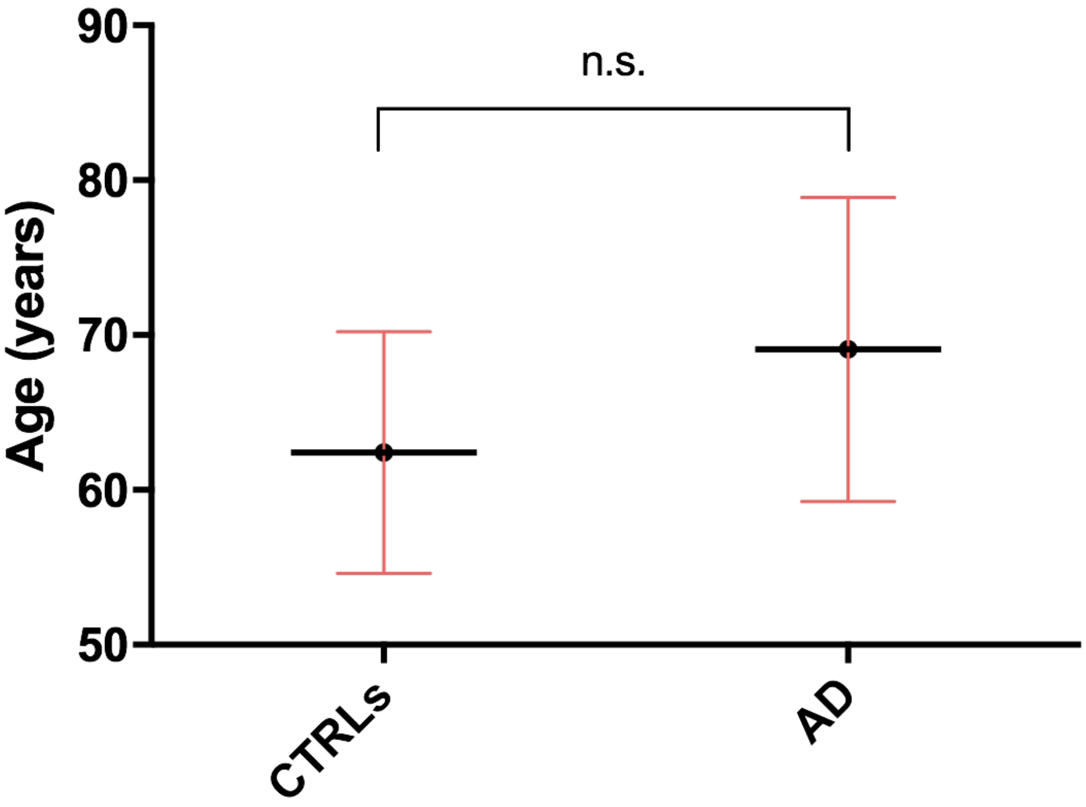

**Figure S8. *HBB* and *HBA1/2* expression levels in females and males.**

$\Delta C_T$ values were normalized against *GAPDH* and *ALAS2*. F female, M male

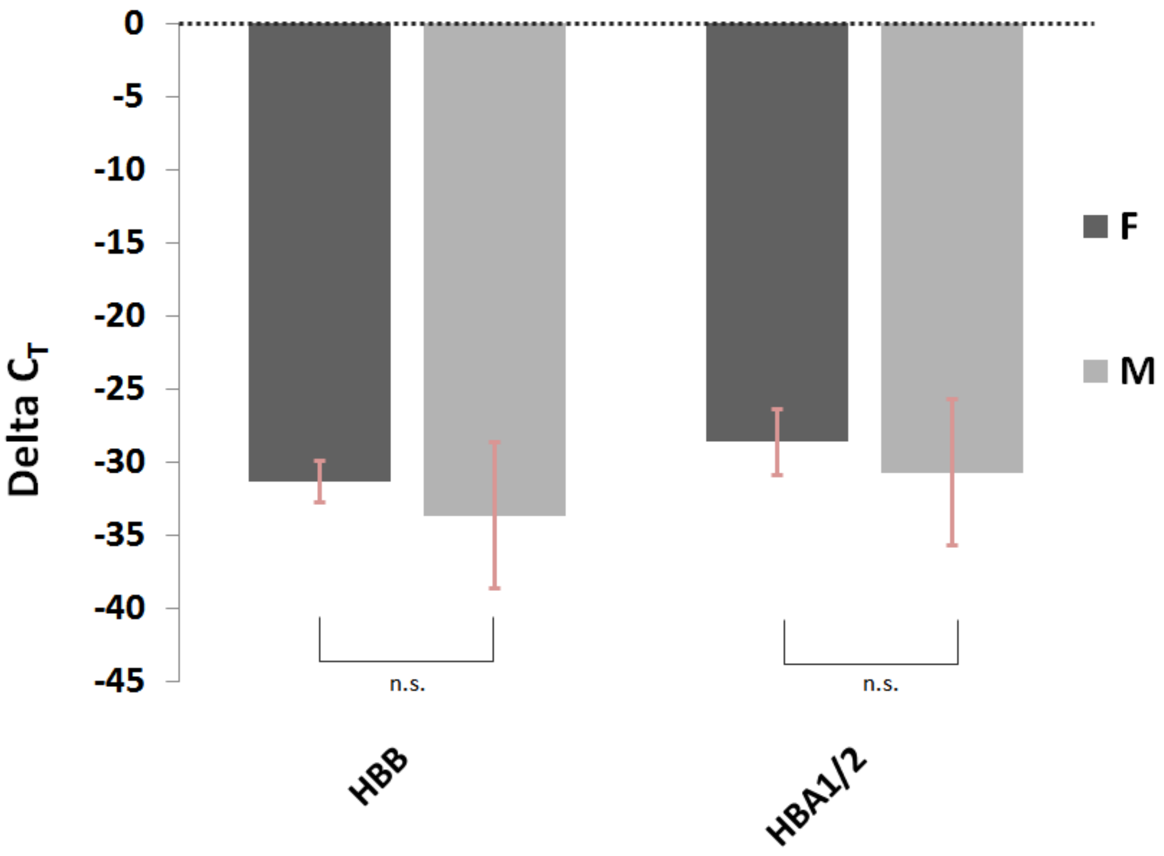

**Figure S9.** ***HBB*** **and** ***HBA1/2*** **expression levels across healthy controls.**
$\Delta C_T$ values were normalized against *GAPDH* and *ALAS2*. Age of each single patient is listed on the X axis.

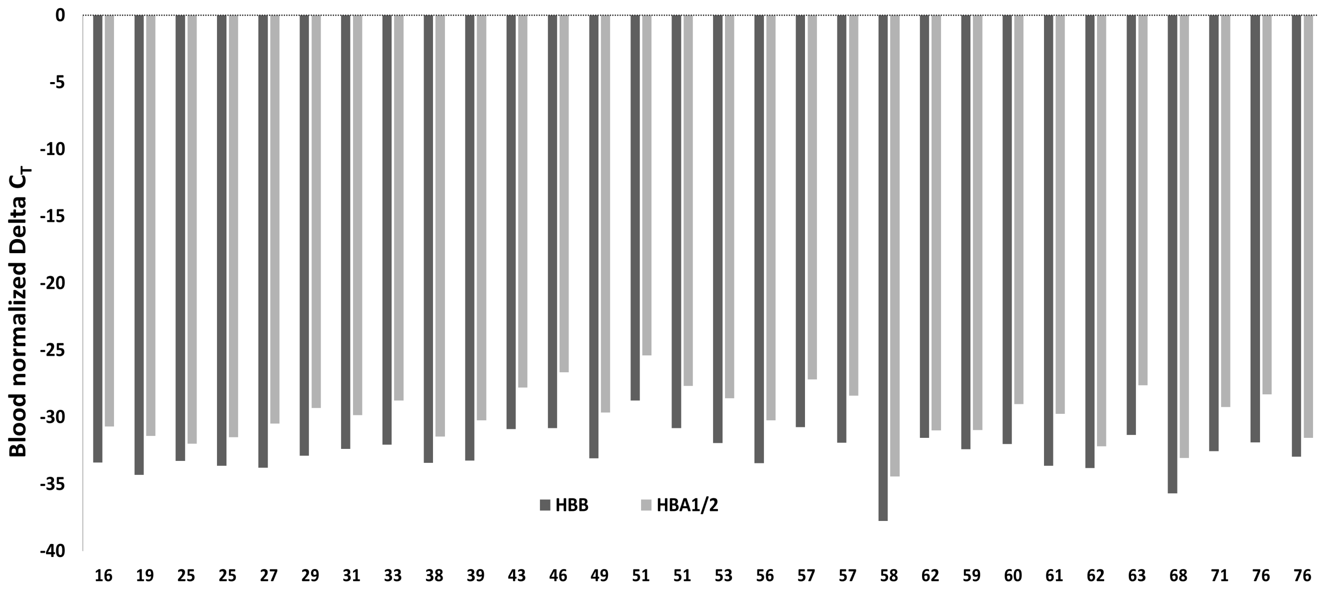

**Figure S10. Hemoglobin α-chain (A, B, C) and β-chain (D, E, F) in the frontal cortex of a single sCJD case.** Immunofluorescence and confocal microscopy for hemoglobin α-chain (A), β-chain and nuclei (B, E) showing increased immunoreactivity in astrocytes for α-chain hemoglobin (C merge) but not for . β-chain (F merge) Nuclei staned with DRAQ5™. Bar = 50 μm.

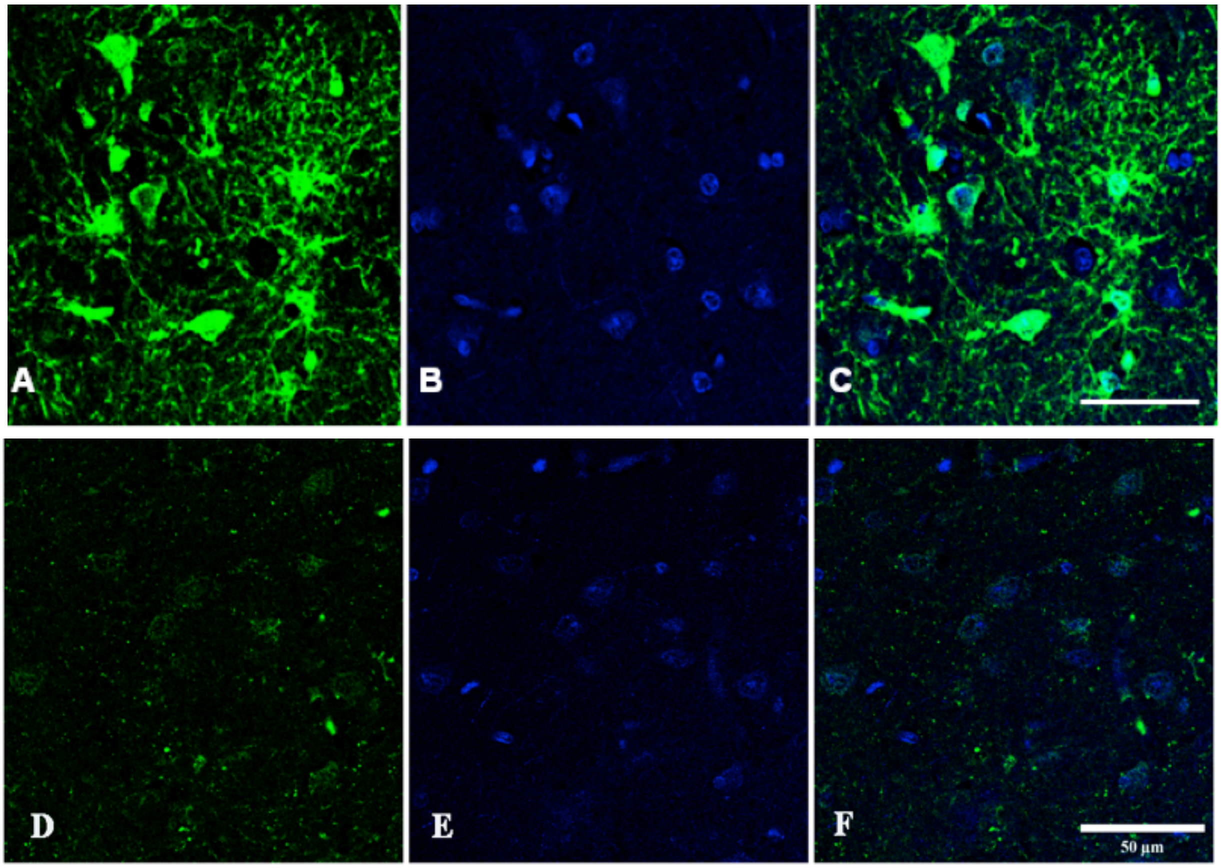

**Figure S11. Double-labelling immunofluorescence in the frontal cortex of a single sCJD case.**

A-C) Double-labelling immunofluorescence and confocal microscopy showing haemoglobin A (green, A) and glial fibrillary acidic protein (red, B) in the cerebral cortex in one case with Creutzfeldt-Jakob's disease. Haemoglobin is found in many astrocytes (arrows, C merge). D-F) Double-labeling immunofluorescence and confocal microscopy showing haemoglobin B (green, D) and NeuN (red, E). Haemoglobin is found in most if not all neurons (arrowheads, F merge). Paraffin sections; Bar = 40 microns.

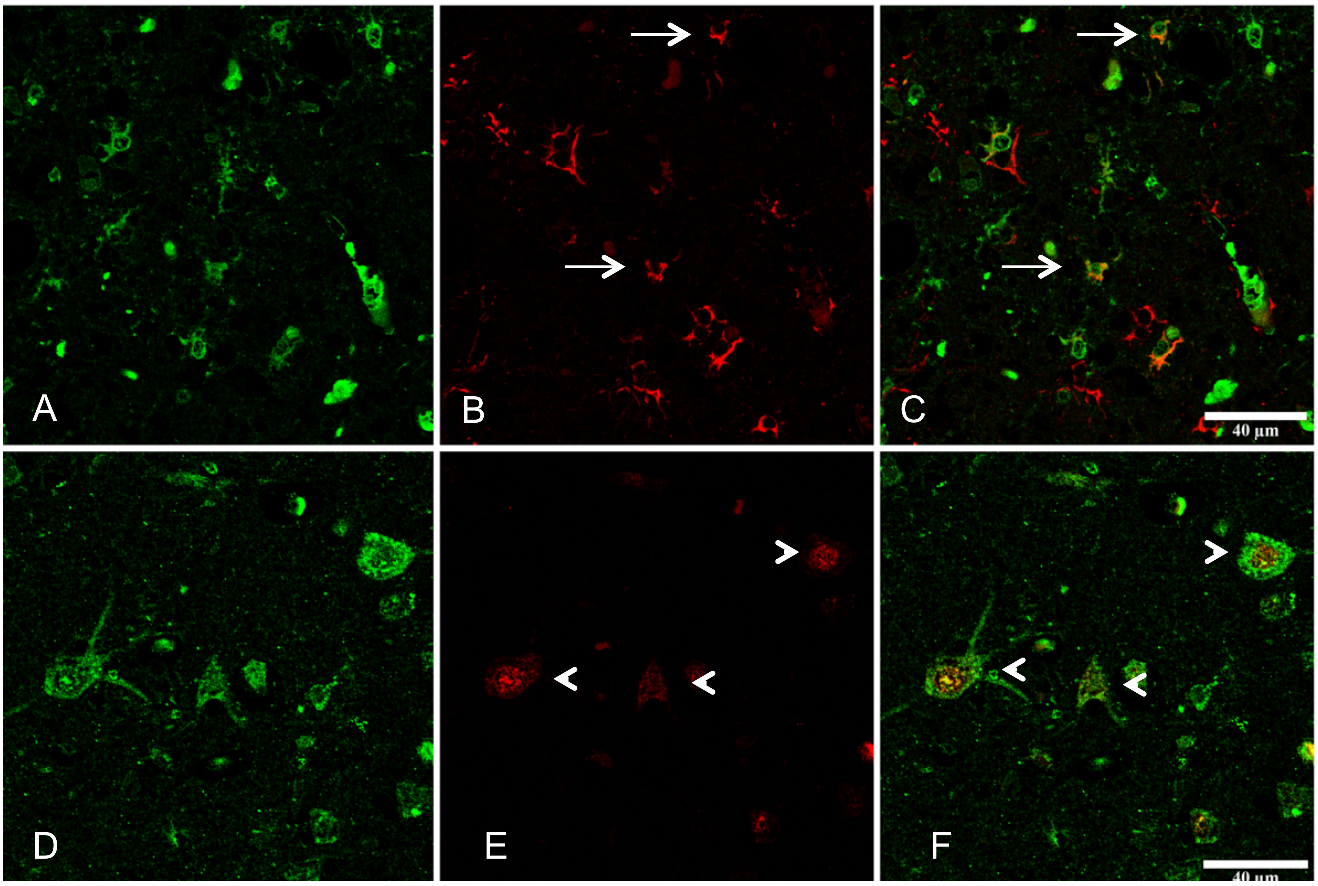

**Table S1. List of AD and healthy controls.** F female, M male, BS Braak stage

| AD | SEX | AGE | BRAAK STAGE | CTRLs | SEX | AGE |
|---|---|---|---|---|---|---|
| A10/46 | M | 74 | AD, BS II | 4176 | M | 51 |
| A11/13 | M | 70 | AD, BS I | 15221 | M | 53 |
| A10/64 | M | 86 | AD, BS II | 3783 | M | 56 |
| A10/45 | M | 67 | AD, BS I | 24781 | M | 57 |
| A10/6 | M | 57 | AD, BS II | 18391 | M | 58 |
| A11/75 | M | 61 | AD, BS I | 7628 | M | 60 |
| A10/98 | F | 73 | AD, BS I | 22612 | M | 61 |
| A11/51 | M | 58 | AD, BS I | 18407 | M | 62 |
| A10/27 | M | 68 | AD, BS I | 20121 | M | 63 |
| A10/77 | M | 65 | AD, BS II | 13410 | M | 68 |
| A11/55 | M | 60 | AD, BS II | 14395 | F | 71 |
| A10/34 | M | 64 | AD, BS I | 9508 | M | 76 |
| 1677 | M | 90 | AD, BS III | 1656 | F | 62 |
| 1721 | M | 74 | AD, BS I | 17/14 | M | 76 |
